# Influence of Low-pH Beverages on the Two-Body Wear of CAD/CAM Monolithic Materials

**DOI:** 10.3390/polym13172915

**Published:** 2021-08-30

**Authors:** Nicola Scotti, Andrei Ionescu, Allegra Comba, Andrea Baldi, Eugenio Brambilla, Alessandro Vichi, Cecilia Goracci, Raffaele Ciardiello, Andrea Tridello, Davide Paolino, Daniele Botto

**Affiliations:** 1 Dental School Lingotto, Department of Surgical Sciences, University of Turin, 10126 Turin, Italy; alle_comba@yahoo.it (A.C.); andrea.baldi@unito.it (A.B.); 2 Oral Microbiology and Biomaterials Laboratory, Department of Biomedical, Surgical and Dental Sciences, University of Milan, 20122 Milano, Italy; AndreiIonescu_40@hotmail.com (A.I.); eugenio.brambilla@unimi.it (E.B.); 3 Dental Academy, University of Portsmouth, William Beatty Building, Hampshire Terrace, Portsmouth PO1 2QG, UK; alessandro.vichi@port.ac.uk; 4 Department of Medical Biotechnologies, University of Siena, 53100 Siena, Italy; cecilia.goracci@unisi.it; 5 Department of Mechanical and Aerospace Engineering, Politecnico di Torino, 10129 Turin, Italy; raffaele.ciardiello@polito.it (R.C.); andrea.tridello@polito.it (A.T.); davide.paolino@polito.it (D.P.); daniele.botto@polito.it (D.B.)

**Keywords:** two-body wear, acidic pH, CAD/CAM materials, roughness

## Abstract

The aim of this in vitro study is to evaluate the effect of different acidic media on volumetric wear and surface roughness of CAD/CAM monolithic materials. Forty-eight rectangular specimens were prepared using different CAD/CAM monolithic materials: nanohybrid composite (Grandio Blocks, Voco), resin-based composite (Cerasmart, GC), lithium disilicate (E-Max, Ivoclar), and high-translucency zirconia (Katana STML, Kuraray Noritake). After storage in distilled water at 37 °C for two days, the specimens were tested using a chewing machine with a stainless-steel ball as an antagonist (49N loads, 250,000 cycles). Testing was performed using distilled water, Coca-Cola, and Red Bull as abrasive media. Wear and surface roughness analyses of the CAD/CAM materials were performed using a 3D profilometer and analyzed with two-way analysis of variance and post hoc pairwise comparison procedures. Worn surfaces were examined using scanning electron microscopy. Resin-based materials suffered higher volumetric wear than ceramics (*p* = 0.00001). Water induced significantly less volumetric wear than the other tested solutions (*p* = 0.0014), independent of the material tested. High-translucency zirconia showed less surface roughness than all the other materials tested. The selection of monolithic CAD/CAM materials to restore worn dentition due to erosive processes could impact restorative therapy stability over time. Resin-based materials seem to be more influenced by the acidic environment when subjected to a two-body wear test.

## 1. Introduction

The continuous and constant development of digital technologies has led to the affirmation of restorative and prosthetic restorations obtained through computer-aided design and computer-aided manufacturing (CAD/CAM) processes. This development has led to the increasingly frequent use of monolithic materials produced from a single blank, either ceramic or resin-based, produced from a single block. Currently, as stated in the classification by Gracis et al. [1], restorative materials can be grouped into three main families: glass matrix ceramics, polycrystalline ceramics, and resin matrix ceramics. All of these could be employed to restore partially to heavily compromised teeth after big carious lesions, fractures, or extensive wear. 

Ceramic materials are known for their optimal biocompatibility, strength, high esthetics, low plaque accumulation, low thermal conductivity, high color stability properties, and characteristics similar to human enamel. However, the disadvantage of dental ceramics is that they can cause increased wear of the opposing enamel compared with other restorative materials in general [2]. Additionally, traditional composite resins show poor mechanical properties and low wear resistance to mechanical forces [3]. However, the newly developed nanohybrid composite resin-based materials possess high resistance to wear because they contain fillers of various hardness and sizes that enable them to withstand the masticatory forces generated by posterior teeth [4,5]. The more recently introduced resin-based ceramics and hybrid ceramics—thanks to their high inorganic content, high temperature, and pressure polymerization process—showed consistent chemical and mechanical properties [6,7,8], so they are considered a valid option for monolithic adhesive restorations.

The ideal restorative material should fully resemble tooth hard tissues that it replaces, both from mechanical and esthetical viewpoints. For example, ceramics and enamel wear through a similar microfracture mechanism, while composite resins wear through fatigue and abrasion [2,9]. It has been reported that, within ceramics, lithium disilicate has higher wear resistance and causes less wear on opposing enamel compared with conventional feldspathic porcelain [10]. However, oxide ceramics, zirconia in particular, are popular because of their excellent biocompatibility and high strength, and they show more wear resistance than other dental ceramics and restorative materials [11].

The oral cavity is a complex environment in which restorative materials are subjected to severe chemical and physical stresses due to temperature changes, different functional and parafunctional loads, and chemicals from food and drinks [12]. In today’s patients, dental wear is a common cause of tooth damage, with different anthological factors ranging from a superficial loss of enamel surface to complete dentin exposure [13]. Dental erosion, defined as the pathological, chronic, and irreversible dissolution of dental hard tissues caused by acids of a nonbacterial origin, was identified as a globally emerging oral health problem [14]. Dental erosion can cause dentinal hypersensitivity, aesthetic concerns, and loss of vertical dimension—all of which affect oral health related to quality of life [15,16].

In vitro studies have shown that sweet carbonated drinks, sports beverages, and fruit juices cause a loss of hardness in the enamel, presumably because they contain acids (carbonic acid, phosphoric acid, malic acid, and citric acid) [17], which, owing to their low pH, weaken the link between calcium and the phosphate mineral composition of the enamel and dentin, causing mineral loss [18]. Today, the treatment approach toward worn dentition is represented by additive adhesive direct or indirect restorations, aiming to replace tooth structure loss with minimal preparations, thus reducing the further mutilation of enamel and dentin. This is possible owing to the improvement of adhesive systems, which are more user-friendly and stable over time [19,20,21], and restorative materials obtained through digital workflows. However, similar to enamel, some acidic beverage formulations can also induce surface degradation and the increased wear of composite materials [22]. In addition, the acidic environment could induce aqueous corrosion of ceramic glasses because of the selective leaching of alkali ions [23]. 

Today, various material solutions are available to clinicians to decide on the best option, depending on the patients’ oral conditions. Thus, to provide long-term restorations to worn-tooth patients due to erosive processes, materials should demonstrate good wear resistance to the softening effects of the chemical environment. Therefore, the aim of this in vitro study is to investigate the cumulative effects of acidic soft drinks on the wear rate and roughness of different CAD/CAM restorative materials by simulating the oral environment in vitro. The null hypotheses tested are that volumetric wear and surface roughness are not influenced by (1) the CAD/CAM monolithic material and (2) the acidic beverages.

## 2. Materials and Methods

### 2.1. Study Design

This study was designed with 48 samples divided into four study groups (*n* = 12 each), where the specimens were randomly allocated, considering the following:

- “CAD/CAM monolithic material” in four levels: four different materials, commonly employed for worn dentition rehabilitation, were selected: nanohybrid CAD/CAM composite resin (NC, GrandioBlock, Voco GmbH, Cuxhaven, Germany), resin-based composite (RBC, Cerasmart 270, GC Corporation, Tokyo, Japan), lithium disilicate (LD, E-max CAD, Ivoclar, Shaan, Luxembourg), and high-translucency zirconia (ZR, Kuraray Noritake, Tokyo, Japan) (Table 1).
- “Acidic beverage” in three levels: a two-body wear test was performed with samples immersed in three liquid mediums: distilled water, Coca-Cola, and Red Bull.

### 2.2. Sample Preparation

Four different CAD/CAM materials, listed in Table 1, were selected for this in vitro study: Cerasmart 270, shade A2 LT (GC Corporation, Tokyo, Japan); Grandio Block, shade A2 LT (Voco GmbH, Cuxhaven, Germany); E-Max CAD, shade A2 LT (Ivoclar, Shaan, Luxembourg); Katana STML, shade A2 (Kuraray Noritake, Tokyo, Japan). The same shade was selected for all samples (Vita A3). Specimens were obtained by cutting CAD/CAM blocks to a thickness of 2 mm with a low-speed diamond saw (Micromet, Rockville, MD, USA). After cutting, LD and ZR were crystallized and sintered with Cerec SpeedFire, according to the manufacturer’s instructions, and then embedded in the center of a circular stainless-steel mold with a light-curing resin. Subsequently, all specimens were polished with metallographic SiC paper (600, 800, 1200, and 2400 grit) and subsequently cleaned in distilled water for 5 min in an ultrasonic bath. Samples were stored for seven days at 37 °C before the two-body wear test with a chewing simulator. The simulator was run, and the specimens were digitized by a single operator (A.B.) to ensure standardized handling.

### 2.3. Wear Simulation Test

CAD/CAM material specimens were mounted on a four-chamber dual-axis chewing simulation (CS 4.4; SD Mechatronic GmbH, Feldkirchen-Westerham, Germany) after randomly dividing them into three subgroups according to the liquid medium in which the wear test was performed: distilled water (subgroup A), Coca-Cola (subgroup B), and Red Bull (subgroup C). All samples were aged with 250,000 cycles in “low impact mode,” with a vertical load of 49 N, a frequency of 1.2 Hz, and a sliding horizontal movement of 2 mm. As an antagonist, a new 2 mm diameter steel-metal point was employed for each specimen. 

### 2.4. Sample Scan and Analysis

An optical system (Alicona IFM G4g) based on the technology “Focus-Variation” was used for a tri-dimensional (3D) survey of the contact surfaces of the tested materials. With focus variation technology, the contact surface to be measured was illuminated by white light, and the operating principle combined the small depth of focus of an optical system with vertical scanning. The selection of both vertical and lateral resolutions can be realized through a simple change of objectives. In this work, surfaces were measured with an objective 5x. The large dimensions of the surface advised against higher magnification that, despite a better resolution, would have produced a huge output file, which is difficult to manage during the post-processes phase. The 5x objective gives vertical and optical lateral resolutions of 0.4 and 2.2 μm, respectively. Surfaces were measured before starting the chewing test. This surface will be referred to as the “new” surface. The “worn” surface was measured after the completion of the planned number N of chewing cycles. A reference surface was assessed by identifying the portion of the surface out of the contact region to calculate the volumetric wear. This surface is defined as an unworn surface. The form of the unworn surface is assumed a priori. In this work, the form was assumed to be a plane. The coefficients defining the form are obtained with the best fit by minimizing the error between the unworn surface and the form with the least squares method. The fitting was performed by excluding the worn area. The volume loss was then determined by taking the difference between the form and the worn surface using dedicated software (IF-MeasureSuite 5.1, Bruker Alicona, Graz, Austria).

Using the same software, the surface roughness of the worn area of all specimens was calculated. Five linear measurements inside the steel-metal point scratch, perpendicular to the sliding direction of the two-body wear test, were performed, and the mean R_a_ values were calculated to obtain the surface roughness per specimen. 

### 2.5. SEM Analysis

**Figure 1:**
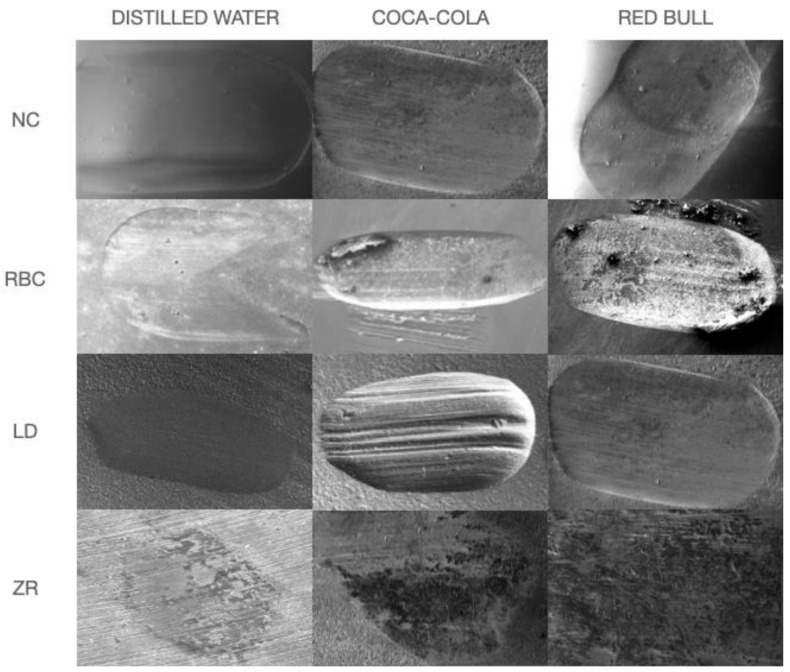
Representative scanning electron microscope images of the study specimens. It is evident how specimens immersed in acidic pH beverages during chewing simulation showed a different worn surface from water-immersed ones. All materials showed a rough surface with grooves oriented parallel with the sliding direction, indicating an abrasive wear mechanism. ZR revealed wear pits associated with the dislodgment of ceramic particles on the worn surfaces of the zirconia ceramics.

After the quantitative wear evaluation, the abraded samples were sputter-coated with gold and observed under a scanning electron microscope (SEM) (EVO 50 XVP LaB6, Carl Zeiss SMT Ltd., Cambridge, UK) at 503 magnifications to analyze the wear facets produced throughout the chewing simulation. SEM conditions were set as follows: high vacuum (2_10_7 Torr), emission current 10 pA, accelerating voltage 10 kV, and working distance around 10 mm (Figure 1).

### 2.6. Statistical Analysis

A Shapiro–Wilk test revealed that the data were normally distributed. To examine the effects of the factors “material” and “acidic beverage” and their interactions on volumetric wear and surface roughness, a two-way analysis of variance test (ANOVA) was conducted. Post hoc pairwise comparison was performed using Tukey’s test. All statistical analyses were performed using STATA software (ver. 12.0; StataCorp, College Station, TX, USA).

## 3. Results

The mean volumetric wear (±SD, expressed in mm^3^) is summarized in Table 2. Two-way ANOVA reported a significant influence of the factor “material” (*p* = 0.00001) and of the factor “acidic beverage” (*p* = 0.0014) but not for their interaction (*p* = 0.52). Tukey’s post hoc test showed that RBC and NC suffered greater volumetric wear than LD and ZR, showing a statistical significance between them. Regarding acidic beverages, distilled water induced less volumetric wear than the other tested solutions.

The mean surface roughness (±SD, expressed in R_a_ and measured in μm) is summarized in Table 3. Two-way ANOVA reported a significant influence of the factor “material” (*p* = 0.00001), while no significant differences were reported for the “acidic beverage” factor (*p* = 0.22), and their interactions (*p* = 0.14) were shown. Tukey’s post hoc test reported that ZR showed inferior surface roughness compared with other tested materials. Moreover, both RBC and NC showed greater R_a_ than LD.

## 4. Discussion

The first null hypothesis was rejected based on the obtained results since the CAD/CAM monolithic materials tested showed significantly different volumetric wear and surface roughness after the two-body wear test. However, different acidic beverages significantly influenced volumetric wear but not surface roughness; thus, the second null hypothesis was partially accepted.

The evaluation of wear is not a simple task, especially in mild wear conditions where the amount of material removed is small, and the volume loss could be the same order of magnitude as the measurement error. In this study, two methods were used to evaluate volume loss: (i) a direct comparison between the new and the worn surface and (ii) a comparison between the worn surface and a “reference surface”. The direct comparison is more intuitive and seemingly simpler, but it is not appropriate when small volumes of wear are involved. It is complex to overlap the 3D scan of the worn surface with the 3D scan of the new surface with the desired accuracy, because neither the specimen nor the measuring instrument has reference points or markers that allow overlapping the two measured surfaces with the needed accuracy. In the second method, a reference surface is defined using a portion of the surface that has not been involved in the wear process, such as a portion of the surface out of the contact region. This surface is defined as the unworn surface. The form of the unworn surface is assumed a priori. In this study, the form was assumed to be a plane. The coefficients defining the form are obtained with the best fit by minimizing the error between the unworn surface and the form with the least squares method. The fitting was performed by excluding the worn area; therefore, it is reasonable to assume that the form obtained with the best fitting procedure is an accurate approximation of the new surface. The volume loss is then determined by taking the difference between the form and the worn surface [24].

This study evaluated the effect of low-pH soft drinks on monolithic material wear and roughness; thus, a two-body wear test was conducted. Indeed, the sliding movement was performed between the stainless-steel point and the specimens’ surfaces while immersed in water, Coca-Cola, or Red Bull. The laboratory setup was intended to simulate the acidic environment of the oral cavity by an uninterrupted immersion of the specimens in acidic beverages. A limitation of this in vitro model was that other oral cavity factors that could influence the ecosystem, such as salivary buffering capacity or acquired pellicle, were disregarded [25]. However, previous studies have shown how uninterrupted immersion in acidic drinks is a valuable in vitro simulation condition to test composite dental materials [26,27]. 

The erosive potential of low-pH soft drinks toward tooth structure is well known and widely studied [28]. A recent review [29] stated that carbonated drinks were significantly positively associated with dental erosion in 52% of studies that investigated these beverages. In this context, the consumption of an acidic diet could directly impact hydroxyapatite dissolution and could also occur for dental materials. Indeed, the durability of restorations in the oral cavity is highly affected by the resistance to dissolution or disintegration caused by foods, drinks, and the acidity produced by bacteria [26]. The pH values of Coca-Cola (2.34–2.96) and Red Bull (3.28–3.43) [30] were lower than the critical pH for enamel demineralization, but they could be considered critical for restorative materials as well. Previous studies have shown that the persistence of an acidic pH contributes to the degeneration of materials’ properties [31,32]. It is known that restorative materials can absorb water and other acidic fluids, causing surface degradation. Previous studies have found that water could act as a conductor for acidic penetration into the resin matrix of composites. Badra et al. [22] revealed that the microhardness of materials immersed in Coca-Cola remained stable for up to 7 days but showed a decrease after 30 days. 

In this study, the tested beverages increased the volumetric wear of RBC and NC. Water molecules can induce the degradation of composites via two mechanisms. First, they diffuse into the polymer network and occupy the free volume between polymer chains and microvoids, causing plasticization and swelling of the polymer matrix and initiating chain scission, causing monomer elution [33,34]. Mayworm et al. [35] stated that in the oral cavity, the softening of dental composite resin matrices by saliva probably aggravates the undesirable effects of wear during mastication and tooth–tooth contact. Thus, the association between saliva sorption and wear promotes a cyclic effect: saliva softens the restoration of the superficial layer, which can be more easily removed by wear. In this context, it could be speculated that soft drinks, due to their higher density and acidic pH, which could promote the dissolution of a material surface submitted to cyclic force, could enhance the wear rate of dental materials. In the rolling and sliding of polymers, each asperity of the rubbing surface experiences cyclic stress from the asperities of the counterface. Thus, stress cyclic and plastic strains accumulate, and multiple surface and subsurface cracks are ultimately initiated. With further cycling, these cracks propagate deeply into the substrate, or join their neighbors until one crack becomes large enough to break from the bulk, causing pitting and spalling. The process continues, resulting in a progressive loss of material from the polymer worn surface. The nature and number of crack initiation sites on the surface depend on the loading type and sliding conditions, frequently resulting in larger wear debris. In this context, the low-pH environment can impact the wear rate of RBC and NC, as shown in this study and the previous studies, reporting the wear effects of acidic beverages on different resin composite materials. 

However, this study showed that monolithic CAD/CAM ceramics are less susceptible to volumetric wear than resin-based materials. Among them, ZR exhibited a significantly lower volumetric reduction than LD. The lower wear resistance of lithium disilicate compared with zirconia is attributed to its lower hardness and higher susceptibility to slow crack growth (stress-corrosion) and lower fatigue threshold [36], and surface corrosion (corrosion wear). In a three-year clinical study, CAD/CAM-generated composite crowns showed preservation of the occlusal anatomic form of only 26.5% versus 96% for ceramic crowns [37]. Notwithstanding the recent structural improvements in RBC materials, their lower resistance toward abrasive wear mechanisms than ceramics could still be an issue in the rehabilitation of bruxist patients with VDO reduction. Furthermore, Mormann et al. [38] showed how CAD/CAM resins are more susceptible to volumetric wear but more respective toward antagonist enamel. Another thing to consider in the results of this study is the correspondence between wear and surface roughness. As observed in this study, monolithic CAD/CAM ceramic materials generally exhibited lower surface roughness than resin-based ones. Although it is clear how to guarantee long-term clinical success [39], all restorative materials should be sufficiently smoothed by post-grinding processes, such as polishing and glazing. Smooth and polished surfaces support the esthetic appearance of dental restorations, minimize biofilm formation, bacterial adhesion [40,41], fatigue, chipping, or fracture [42], and improve flexural strength [43]. Moreover, as previously mentioned, surface roughness is a critical aspect of the wear mechanism, with smooth surfaces undergoing less wear [44,45] and extending the restoration’s longevity. Thus, it is important to mention how the clinician should pay attention to the polishing of CAD/CAM restorative materials to minimize the volumetric wear of the restorative material, above all, for polymers such as RBC and NC. In this study, it should be specified that optical roughness measurements using a 3D laser scanning microscope were performed. Optical roughness testing allows for measuring a smaller asperity than contact types, and repeated tests can be performed without surface scratching. However, the surface reflection might influence the evaluation, and the results may differ from contact surface data. Another limitation of the study was represented by how the two-body wear test was conducted, which did not allow us to properly understand the role of the acidic challenge alone in volumetric wear and surface roughness. Further studies could be necessary to better understand the role of the acidic pH of soft drinks with and without simultaneous mechanical sliding to evaluate eventual chemical interactions with the polymers’ matrix of resin-based materials.

## 5. Conclusions

Within the limits of this study, it was observed that low-pH soft drinks impact the volumetric wear of CAD/CAM resin-based materials, while lithium disilicate and high-translucency zirconia were more resistant to an abrasive mechanism. 

## Figures and Tables

**Table 1:** Manufacturer, classification and composition of tested materials.

Name	Manufacturer	Classification	Composition (*)
Grandio Blocs (NC)	VOCO GmbH, Cuxhaven, Germany	Nanohybrid Composite	86% *w/w* of and glass-ceramic fillers, functionalized silicon dioxide nanoparticles, Bis-GMA, UDMA, TEGDMA
Cerasmart 270 (RBC)	GC Dental Products, Tokyo, Japan	Resin-based composite	71% *w/w* of barium and silica nanoparticles, Bis-MEPP, UDMA, dimethacrylate
E-Max CAD (LD)	Ivoclar Vivadent, Shaan, Luxemburg	Lithium Disilicate	SiO2 60–65%; K2O 15–19%; Al2O3 6–10.5%; other oxides and pigments 0–8%
Katana ML (ZR)	Kuraray Noritake, Tokyo, Japan	High-translucency Zirconia	Zr 60%, O 30%, Hf 1.3%

(*) Bis-GMA = Bisphenol A glycidyl methacrylate, TEGDMA = triethylene glycol dimethacrylate, Bis-EMA = Bisphenol A ethoxylated methacrylate, UDMA = urethane dimethacrylate, DX-511 = High molecular weight Dupont monomer, Bis-MEPP = Bisphenol A ethoxylate dimethacrylate.

**Table 2:** Volumetric wear, expressed as mean ± standard deviation for all the tested subgroups. Same superscript capital letters indicate no difference between row results. Same superscript lower-case letters indicate no difference between column results.

	Volumetric Wear (mm^3^)		
	Water	Coca-Cola	Redbull
RBC	0.28 ^a,A^ ± 0.08	0.34 ^a,B^ ± 0.11	0.34 ^a,B^ ± 0.09
NC	0.29 ^a,A^ ± 0.06	0.36 ^a,B^ ± 0.07	0.36 ^a,B^ ± 0.04
ZR	0.01 ^c,A^ ± 0.003	0.02 ^c,A^ ± 0.02	0.02 ^c,A^ ± 0.02
LD	0.16 ^b,A^ ± 0.09	0.20 ^b,A^ ± 0.08	0.21 ^b,A^ ± 0.09

**Table 3:** Surface roughness, expressed as mean ± standard deviation, for all the tested subgroups. Same superscript capital letters indicate no difference between row results. Same superscript lower-case letters indicate no difference between column results.

	Surface Roughness R_a_ (µm)		
	Water	Coca-Cola	Redbull
RBC	2.08 ^a,A^ ± 0.33	2.43 ^a,A^ ± 0.34	2.43 ^a,A^ ± 0.92
NC	2.50 ^a,A^ ± 0.20	3.04 ^a,A^ ± 0.79	3.40 ^a,B^ ± 0.70
ZR	1.49 ^b,A^ ± 0.24	1.36 ^b,A^ ± 0.08	1.39 ^b,A^ ± 0.46
LD	1.87 ^b,A^ ± 0.71	1.73 ^b,A^ ± 0.71	1.69 ^b,A^ ± 0.56

## Data Availability

The data presented in this study are available on request from the corresponding author.
